# Accuracy and precision of stimulus timing and reaction times with Unreal Engine and SteamVR

**DOI:** 10.1371/journal.pone.0231152

**Published:** 2020-04-08

**Authors:** Michael Wiesing, Gereon R. Fink, Ralph Weidner

**Affiliations:** 1 Cognitive Neuroscience, Institute of Neuroscience and Medicine (INM-3), Research Centre, Juelich, Germany; 2 Department of Neurology, University Hospital Cologne and Faculty of Medicine, University of Cologne, Cologne, Germany; University of Milan, ITALY

## Abstract

The increasing interest in Virtual Reality (VR) as a tool for neuroscientific research contrasts with the current lack of established toolboxes and standards. In several recent studies, game engines like Unity or Unreal Engine were used. It remains to be tested whether these software packages provide sufficiently precise and accurate stimulus timing and time measurements that allow inferring ongoing mental and neural processes. We here investigated the precision and accuracy of the timing mechanisms of Unreal Engine 4 and SteamVR in combination with the HTC Vive VR system. In a first experiment, objective external measures revealed that stimulus durations were highly accurate. In contrast, in a second experiment, the assessment of the precision of built-in timing procedures revealed highly variable reaction time measurements and inaccurate determination of stimulus onsets. Hence, we developed a new software-based method that allows precise and accurate reaction time measurements with Unreal Engine and SteamVR. Instead of using the standard timing procedures implemented within Unreal Engine, time acquisition was outsourced to a background application. Timing benchmarks revealed that the newly developed method allows reaction time measurements with a precision and accuracy in the millisecond range. Overall, the present results indicate that the HTC Vive together with Unreal Engine and SteamVR can achieve high levels of precision and accuracy both concerning stimulus duration and critical time measurements. The latter can be achieved using a newly developed routine that allows not only accurate reaction time measures but also provides precise timing parameters that can be used in combination with time-sensitive functional measures such as electroencephalography (EEG) or transcranial magnetic stimulation (TMS).

## Introduction

Over the last 20 years, Virtual Reality (VR) has been increasingly recognized as a powerful research tool in behavioral neuroscience [1,2]. VR enables researchers to study complex and naturalistic behavior in virtual environments while maintaining a high degree of experimental control. VR can help to increase the ecological validity of experiments, i.e., allowing to conduct experiments in a context that is closer to everyday life, which might lead to more generalizable and valid explanations regarding cognitive processes. For example, a virtual scenario of a classroom allows to investigate the attentional capacities of children with ADHD in a realistic but well-controlled environment [3].

Consumer-grade HMD based VR Systems like Oculus Rift, Valve Index, or HTC Vive are now making VR available for many researchers and will soon dramatically increase the impact of VR in the field of cognitive neuroscience. Most likely, VR will have major impact on studies of visual perception as one of the many advantages that VR offers is its ability to present stimuli in stereoscopic 3D with a large field of view and with the HMDs entirely covering the visual field.

Yet, the suitability and reliability of HMDs concerning the measurement of visual cognitive performance needs to be demonstrated. Recent studies examined whether HMDs, at least in principle, can be used to reliably investigate visual processing components. The results indicate, that the Oculus Rift Development Kit 2 (DK2) and the HTC Vive allow assessing visual processing as reliably as CRT displays [4,5].

In addition, due to a lack of established stimulus software for VR experiments, several recent studies relied on using game engines like Unity [6] or Unreal Engine [7] [e.g., 8, 9]. However, game engines do not contain certain key features that are critical for neuroscientific experiments. For example, data collection with build-in features, such as response time measurements, are tied to the software’s frame rate, resulting in a sampling rate of 90 Hz when using an HMD such as Oculus Rift CV1, HTC Vive, or HTC Vive Pro.

Furthermore, modern VR systems use runtime software tools like SteamVR [10] or the Oculus Rift Software [11], which operate in the so-called “direct mode” that allows direct communication with the HMD hence bypassing the operating system’s typical display communication pathways. These runtime software tools determine when a new frame will be presented. They thus have control over the exact timing of stimulus presentation, while at the same time limiting the accuracy with which stimulus events can be measured, at least when the game engine’s build-in timing methods are used.

These limitations are well described in the literature [e.g., 12,13] and different approaches have been proposed to increase the precision and accuracy of time critical measurements in combination with game engines [e.g., 14–16].

However, to date, no study has systematically quantified the maximum precision and accuracy of stimulus timing and time measurements achievable with consumer VR hardware in combination with game engines.

The goal of the present study was (1) to assess timing errors of the HTC Vive combined with Unreal Engine 4 (UE4) and SteamVR, and (2) to present a new method that allows to measure the timing of visual stimulus events and response times with an accuracy and precision in the millisecond range.

### Displays

Experimental neuroscience requires accurate and precise control of stimulus durations and is hence in need for display techniques with appropriate temporal properties. Display technologies differ in how accurate stimulus onsets and offsets can be determined. The display technology currently used in VR differs from the computer screens often used in experimental neuroscience. For instance, in contrast to cathode-ray tube (CRT) or liquid crystal displays (LCD) monitors, many modern HMDs such as Oculus Rift or HTC Vive use organic light-emitting diode (OLED) displays, and their accuracy remains to be tested. In particular, it is essential to determine how accurately stimulus onset times (i.e., the time point when a stimulus appears on the display) can be controlled and how precise and solid stimulus durations can be defined.

For a long time, CRT monitors were considered the gold-standard for visual stimulus delivery in experimental neuroscience. In CRT monitors every pixel is updated periodically to generate an image via an electron beam that scans all pixels in rows from top-left to bottom right, a process referred to as raster scanning [17]. An important characteristic here is a pixel’s persistence, i.e., the time pixels are illuminated after stimulation by the electron beam. Each pixel is covered with phosphor. When hit by the electron beam, the phosphor illuminates and quickly reaches its maximum luminance and then starts to fall off again over a given time. This time depends on specific characteristics of the given phosphor and ranges from virtually no persistence (e.g., P15) to several seconds (e.g., P31) [18]. Thus, on a CRT monitor, the entire image cannot be updated simultaneously and does hence not deliver a continuous luminance pattern.

LCD monitors are based on a completely different technology. LCD monitors constitute so-called sample and hold displays in which a steady light source is positioned behind a layer of liquid crystals. The orientation of the crystals defines a pixel’s luminance. Although there is no longer an actual electron beam in modern LCDs, and it is no longer necessary to update displays periodically, it is still common that LCDs are updated in scan like patterns similar to those in CRT monitors. However, in contrast to CRT monitors, LCD signals do not decay and remain constant during the display refresh (except for possible backlight modulations) [17].

Since in both LCD and CRT monitors the image is built up line by line from top to bottom, stimulus onset measurements are synchronized to the vertical blank interval, which is the time between two display refreshes. Therefore, the stimulus onset measurement is only perfectly synchronized to the actual stimulus onset, if the stimulus is presented in the leftmost pixel of the first row. Consequently, the measurement error of the stimulus onset is correlated with the position of the stimulus on the display [19,20].

In contrast, the HTC Vive uses two low-persistence OLEDs. OLEDs are comprised of a thin film of organic material that uses an electrical circuit to control the emission of light [21,22]. Unlike LCDs, OLEDs do not rely on time-consuming reorientations of liquid crystals and have, therefore, faster response times than LCDs. Furthermore, while luminance transition times of LCDs depend on the luminance of the previous frame, in OLEDs, transition times are supposed to be independent of the previous frame’s luminance [23]. In general, OLEDs show precise temporal responses and appear to be suitable for visual neuroscientific research [21] Hence, the displays used in HMD’s are unique with regard to timing and time measurements due to particularities of how the display is refreshed.

The whole display gets loaded before it illuminates so that all pixels illuminate simultaneously. This type of display is hence often referred to as “global displays” [24]. In particular, a display refresh of the HTC Vive’s displays starts with a blank period, during which the displays stay black. The pixels then illuminate for about 2 ms at the end of the screen refresh period. This is done to minimize visual motion artifacts like smearing and judder. Thus, the updating behavior of the HTC Vive’s displays should allow accurate measurements of the stimulus onset, independent of its position.

### Stimulus software

For traditional experiments in visual neuroscience, a variety of commercial and open-source software tools are available that meet strict timing requirements, e.g., Presentation [25], PsychoPy [26], or the Psychophysics Toolbox [27]. These software tools are optimized for the presentation of two-dimensional stimuli and offer no or only limited support for the current state of the art VR systems. Concerning VR, established toolboxes for neuroscientific experiments are lacking. While a variety of commercial and open-source software is available for the creation of virtual environments, most of the software tools do not contain features specifically designed for neuroscientific experiments. One exception is WorldViz Vizard [28], a commercial solution, which allows researchers to create and conduct VR experiments.

However, in several recent studies, game engines like Unity or Unreal Engine were used and it remains to be tested whether these software packages provide precise and accurate stimulus timing and time measurements that allow inference on mental and neural processes.

For instance, a time-critical presentation of visual stimuli is required for visual masking paradigms [29] or attentional blink paradigms [30] as well as for almost any experiment involving visual psychophysics [31]. Similarly, a precise recording of response times is required for all studies involving mental chronometry, with reaction times as a dependent measure. Furthermore, a precise recording of events within an experiment is mandatory for the analysis of all time-sensitive functional measures, such as EEG, TMS, magnetoencephalography (MEG), or galvanic skin response (GSR), where cognitive and perceptual events are assigned to functional markers.

The current study investigated the timing errors of the HTC Vive combined with UE4 and SteamVR. In the first experiment, we assessed the precision and accuracy of stimulus presentations. In the second experiment, we explored the limitations of UE4’s build-in timing procedures for reaction time measurements resulting in variable and inaccurate reaction time measures that are inappropriate for a wide range of reaction time experiments. Further, a newly developed method and its benchmarking results will be presented. This new method allows precise and accurate reaction time measurements with UE4 and SteamVR.

In the following, in order to increase readability, we will always refer to UE4, although, if not stated differently, it always refers to the combination of UE4, SteamVR, and the HTC Vive.

## General methods

### Unreal engine settings

We aimed to keep almost all default settings of UE4 in this study. However, due to the high rendering demands of VR, it is generally recommended to adjust some settings to optimize UE4’s VR rendering.

First, we changed the rendering technique from the default Deferred Renderer to the Forward Renderer. Forward rendering is generally the preferable rendering method for VR since it usually improves performance and allows better anti-aliasing methods than deferred rendering. Forward rendering allows using multisample anti-aliasing (MSAA) that increases sharpness and leads to better visuals [24,32]. Additionally, we enabled Instanced Stereo rendering. By default, the geometry has to be drawn twice for VR applications as compared to non-VR rendering, once for the left eye and once for the right eye, which essentially doubles the number of draw calls, i.e., the rendering information that is send from the central processing unit (CPU) to the graphics processing unit (GPU). With Instanced Stereo rendering, the geometry has to be drawn only once and is then projected to the right-eye view and left-eye view of the geometry. This procedure halves the number of draw calls and thereby saves a substantial amount of CPU time.

All tests described in this paper were conducted with Unreal Engine 4.21.2 with the configuration described above.

### SteamVR settings

In all experiments, we disabled supersampling anti-aliasing (SSAA) by setting the render resolution of the HTC Vive to 100%. SSAA works by rendering a scene with a higher resolution than the one displayed and then averages neighboring samples to create the image [33]. SSAA is often used for VR rendering to reduce aliasing and improve visual quality.

Additionally, we disabled “Motion Smoothing” for all experiments. Motion Smoothing is a method to stabilize the frame rate when an application starts to drop frames. As soon as the frame rate decreases below the HMD’s refresh rate (90 frames per second (FPS) for the HTC Vive), SteamVR reduces the frame rate by half and instead extrapolates every second frame based on the last two presented frames [34]. However, while this method is helpful for the common usage of VR (e.g., gaming) to provide smoothly running VR experiences, it would corrupt all the timing precision and accuracy within a scientific experiment. Thus, it is recommended to disable this option whenever precise timing is required or when the timing of events (e.g., reaction times) needs to be determined precisely. However, besides Motion Smoothing another technique helps to smooth out dropped frames called “Asynchronous Reprojection”. Like Motion Smoothing, Asynchronous Reprojection reduces the number of rendered frames by half. However, instead of extrapolating frames, it repeats the previous frame but reorients it based on the user’s latest head rotation [35]. Since Asynchronous Reprojection cannot be disabled, a VR experiment should always be optimized about hard- and software to ensure stable frame timing.

All tests described in this paper were conducted with SteamVR 1.2.10 with the configuration described above.

## Experiment 1

The aim of Experiment 1 was to test the precision and accuracy of visual stimulus presentations using UE4. The goal was to assess potential discrepancies between the stimulus timing defined by the researcher and the actual stimulus timing on the display. Please note, stimulus duration errors are observed even in software tools that, in contrast to UE4, are especially designed for behavioral experiments [20].

### Material and methods

Tests were performed using the Black Box Toolkit v2 (BBTK) to test the timing precision and accuracy of simple black and white transitions with predefined durations. The BBTK is specially designed for benchmarking these type of tests [36]. We tested three different conditions to determine the precision and accuracy of UE4’s stimulus timing under different rendering workloads. Furthermore, we ran every test on two different Windows computers to compare the timing precision and accuracy on systems with different hardware specifications.

#### Apparatus

All tests were conducted on two different desktop computers with Windows 10 as the operating system. The specifications of Computer 1) were Intel i7-8700, 32 GB DDR4 RAM, and an Nvidia GTX 1080 graphics card, and of Computer 2) Intel i7-7700K, 32 GB DDR4 RAM, and an Nvidia GTX 1080Ti graphics card. The tested VR System was the HTC Vive (1080 x 1200 pixels per eye).

#### General procedure

We used a well-established procedure to test the timing of visual stimulus presentations for PC [37–39]. For each test, a photo-sensor connected to the BBTK was attached to the middle of the HTC Vive’s left lens. The photo-sensor was used to measure the stimulus timing under all conditions with a sampling resolution of 0.25 ms. Stimuli were filled-in squares repeatedly alternating their contrast from black to white. Contrast reversals were programmed to occur after durations of 11.111, 33.333, 66.666, 100, 200, 500, and 1000 ms (i.e., 1, 3, 6, 9, 18, 45, and 90 display refreshes at 90 Hz). All stimulus durations were defined in terms of the frames or ticks.

**Fig 1:**
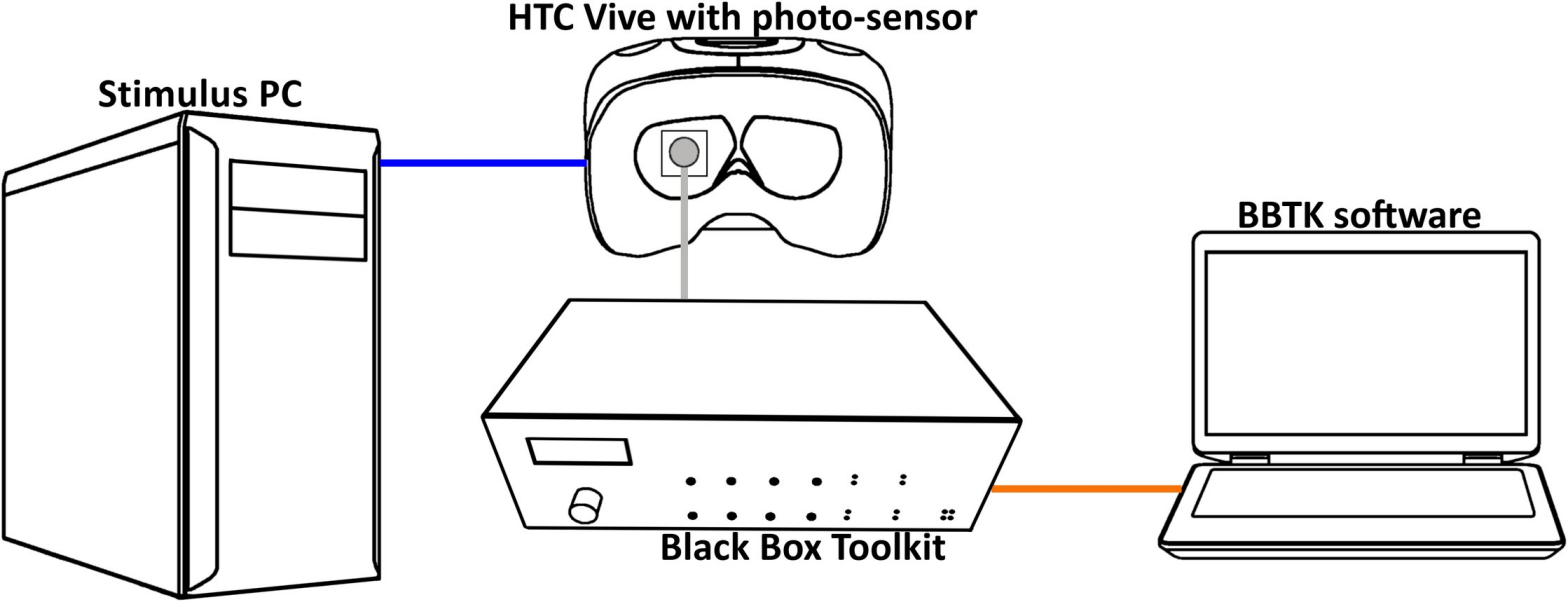
Presentation and measurement setup.

The BBTK was controlled with a second computer that was independent of the presentation computer to prevent interference of the stimulus delivery process and the measurements (Fig 1).

**Fig 2:**
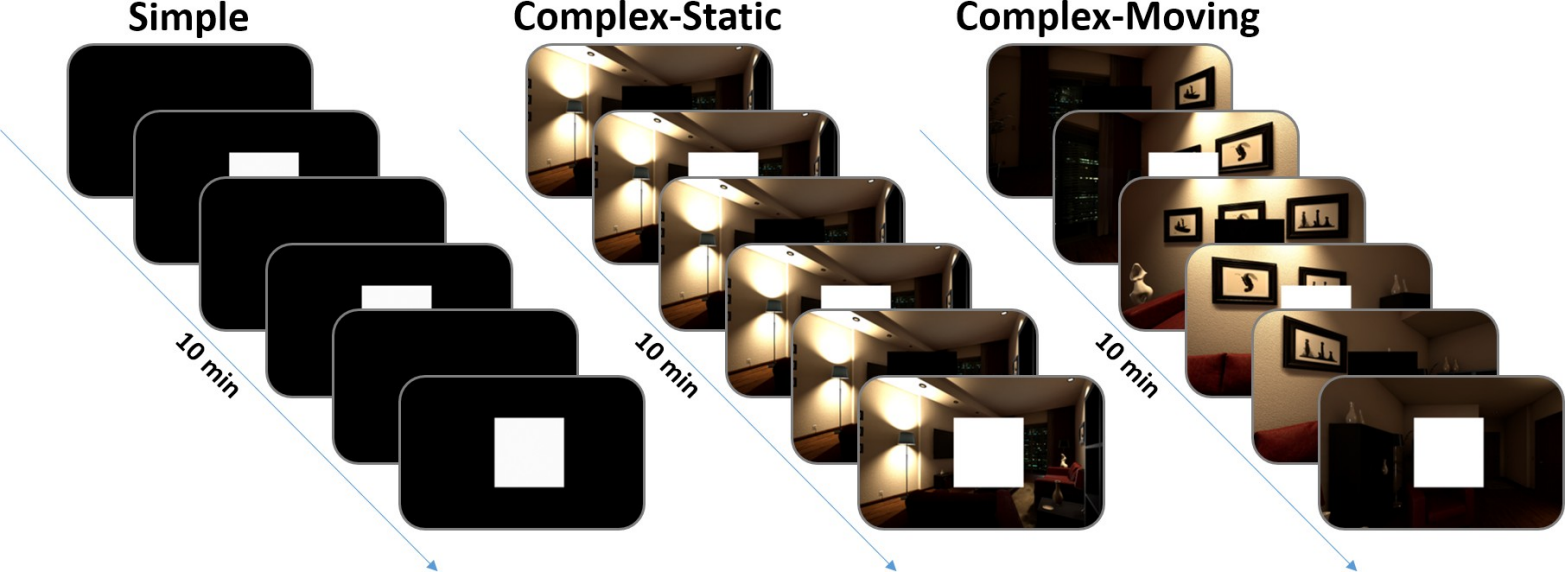
Stimulus conditions. Please note, the brightness of the illustrations was increased and does not represent the actual brightness presented.

Each stimulus duration was tested in a single series with a duration of 10 minutes and hence allowed evaluating the stability of UE4’s timing behavior over long durations. Furthermore, three different conditions were introduced, in which the rendering workload was systematically varied (Fig 2).

To test whether UE4 is generally able to provide sufficient timing precision and accuracy of visual stimulus presentations while the rendering workload is low, a first condition was introduced, which is from now on referred to as *Simple*. In this condition, we created an empty and completely black virtual environment that only consisted of a single square that was presented centrally of the left eye’s image. The square’s brightness alternated between black and white with the durations mentioned above.

This procedure allowed us to determine UE4’s timing precision and accuracy of visual stimulus presentations with a minimal rendering workload and assured that presentation times were unaffected by possible performance constraints.

A second condition, further referred to as *Complex-Static*, aimed to evaluate the stimulus timing precision and accuracy with a high rendering workload. In a typical VR experiment, stimuli are not displayed in isolation but are embedded in a complex virtual environment.

The stimulus material in this condition still consisted of simple black and white transitions. In contrast to the *Simple* condition, the stimulus square was embedded in a virtual environment.

We used the *Realistic Rendering* sample project of UE4 as a virtual environment consisting of a highly realistic and detailed rendered living room [40] (S1 Fig).

A third condition *Complex Moving* aimed to evaluate the stimulus timing precision and accuracy while the movement of a user was simulated. We used the same virtual environment as in the *Complex Static* condition, but of a single static viewpoint, the first-person camera was placed in the center of the environment. The first-person camera was programmed to rotate around the yaw axis with a constant rate of 30° per second. This was done to simulate the user’s head movements. Additionally, to provide a fixed location of the square relative to the camera, we attached the stimulus square to the camera. Apart from these adjustments, the general procedure was identical as in the other conditions.

The displays of the HMD are relatively small, so the photo-sensor could not be directly placed at the stimulus location to detect light from the stimulus square exclusively. Instead, it always included some parts of the surrounding background. Hence, for the *Complex-Static* and *Complex-Moving* conditions, it was necessary to calibrate the photo-sensor’s sensitivity to ensure that the photo-sensor was driven only by the stimulus square and not by the environment’s light.

Calibration consisted of two steps. First, we adjusted the threshold to set the activation point of the BBTK’s photo-sensor to the highest possible value that still detected the white stimulus. In a second step, we adjusted the illumination of the environment. We first replaced the default post-processing of the environment with a custom post-processing. Post-processing describes techniques that change and add visual elements to a rendered scene after the rendering has been completed. This procedure allowed changing the luminance within the scene globally via a single setting, i.e., changing the light exposure, a post-processing effect that changes the overall brightness of a scene. For the calibration, we increased the light exposure until the environment’s light activated the photo-sensor and then reduced it again until the photo-sensor was not activated by it anymore. This procedure ensured a proper illumination of the environment while ruling out any interference between the environment’s light and the photo-sensor’s light measurements.

### Results

For each series, the mean durations of a single stimulus cycle (black and white stimulus interval), as well as the standard deviation and the overall longest and shortest measured durations were computed and reported for all tested stimulus durations. Furthermore, the mean duration of the white stimulus intervals was calculated. However, due to the low persistence displays, the display changed regularly between black and white even during the white stimulus intervals. Accordingly, the BBTK measured constant changes between black and white during white stimulus intervals. Hence, we defined the duration for the white stimulus interval as the time from the first white frame lighting up until the end of the last frame lightning up in that white stimulus interval.

Further, since the low persistence displays only light up for about 2 ms at the end of each display refresh, the white stimulus intervals started with a blank period during which the display was still black. Hence, the BBTK’s photo detector was not able to separate the actual black stimulus interval from this first blank period of the white stimulus intervals first frame. Accordingly, the measured white stimulus interval durations are expected to be shorter than the expected stimulus duration by the duration of one frame’s blank period, i.e., about 9 ms.

The overall variability of the stimulus intervals was consistently within the range of BBTK’s sampling rate of 0.25 ms. However, the measured stimulus durations steadily exceeded the expected, i.e., programmed, stimulus duration. Further, the difference between expected and measured durations appeared to be linearly correlated with the expected stimulus duration. This result was confirmed by a simple linear regression between the expected and measured durations of the stimulus cycles (F(1,5) = 3.62 x 10^16^, p < 0.05), with an R^2^ of 1.

Since the stimulus timing was defined in terms of ticks, the most parsimonious explanation for the difference in stimulus durations was that the true frame rate was lower than the expected 90 FPS. Hence, for all measured stimulus durations, the corresponding frame rate was determined as 89.53 FPS, which was consistent for all tested stimulus durations and computers.

A potential explanation for the lower frame rate could be performance problems affecting the frame rates. However, we systematically varied the rendering workload between conditions and any effect of performance problems on the frame rate should be reflected in different frame rates between conditions.

To test for potential performance problems between conditions, a two factorial Anova with the factors *Condition* (Simple, Complex-Static, Complex-Moving) and *Computer* (Computer 1, Computer 2) was performed. The ANOVA revealed no significant main effect for *Condition* (F(1,2) = 0.001, p = 0.999) indicating no difference between the FPS in the *Simple* (M = 89.528, SD = 0.370), *Complex-Static* (M = 89.528, SD = 0.370), and *Complex-Moving* (M = 89.528, SD = 0.370) conditions. In fact, the values observed in the different conditions were identical up to the fifth decimal place.

Furthermore, potential performance problems should be pronounced differently on the two different computer systems. However, the main effect *Computer* between *Computer 1* (M = 89.529, SD = 0.370) and *Computer 2* (M = 89.527, SD = 0.370) was not significant (F(1,1) = 1.160, p = 0.281). There was also no significant interaction between *Condition x Computer* (F(1,2) = 0.001, p = 0.999).

Besides, to exclude that the reduced frame rate (89.53 Hz vs. 90 Hz) observed was due to particularities of HMD tested, all tests of the *Simple* condition were repeated with an additional HTC Vive system on Computer 1. The mean results of these tests are presented in the supporting information (see S6 Table—S7 Table). The mean frame rate of 89.53 FPS was confirmed with this setup and a one factorial ANOVA with the Factor *HMD* (HMD 1, HMD 2) resulted in a non-significant difference of the mean FPS between *HMD 1* (M = 89.529, SD = 0.370) and *HMD 2* (M = 89.529, SD = 0.370); (F(1,1) = 0.000, p = 0.99).

Table 1 shows the results of the whole set of measurements for the three conditions and two computers. The individual results of the three conditions and both computers separately can be found in the supporting information (see S1 Table–S5 Table).

**Table 1:** Combined results across all conditions and computers (in ms).

expected duration	mean	sd	min	max	mean duration white
**2000**	2010.58	0.116	2010.50	2010.75	996.05
**1000**	1005.29	0.091	1005.25	1005.50	493.41
**400**	402.12	0.125	402.00	402.25	191.82
**200**	201.06	0.105	201.00	201.25	91.29
**133.33**	134.04	0.090	134.00	134.25	57.78
**66.66**	67.02	0.067	67.00	67.25	24.27
**22.22**	22.34	0.120	22.25	22.50	1.93

The first column represents the expected duration for the black and white stimulus cycles. The last column represents the measured durations of the white stimulus intervals.

### Discussion

Experiment 1 aimed to determine Unreal Engine’s timing precision and accuracy for stimulus presentations. Stimulus timing was measured under different rendering workloads. The results revealed that Unreal Engine in combination with the HTC Vive and SteamVR was able to achieve precise and accurate stimulus timings. Timing precision and accuracy were stable even with a high rendering workload. Furthermore, timing precision and accuracy remained stable when the user’s head movements were simulated.

The HMD’s empirical frame rate was determined as 89.53 FPS, slightly differing from the specifications given in the HTC Vive manual. Importantly, the frame rate was stable across different rendering workloads and when tested in combination with different computers, indicating that the observed frame rate was device-specific and not an artifact resulting from potential performance problems. Moreover, additional tests with a second HTC Vive system replicated this finding hence confirming that the reduced frame rate was not the results of a defective HMD.

The results suggest that the true refresh rate of the HTC Vive is 89.53 Hz rather than 90 Hz, at least when used in combination with UE4 running on a Windows 10 platform. Different frame rates may arise when using different software packages. Future research needs to investigate whether these findings hold for different rendering engines and operating systems.

Furthermore, the results of the present study emphasize that when using HMDs such as the HTC Vive, for precise stimulus timing, researchers need to take into account the peculiarities of low persistence displays with the so-called global update. In these displays, every frame starts with a blank period in which the displays stay black. Only after this blank period, the displays illuminate and the image appears. Therefore, black and white flickering stimuli as used in the present experiment involve longer black than white periods. Consequently, studies that rely on exact stimulus timing should consider this difference, when specifying stimulus durations.

Overall, we conclude that UE4, together with SteamVR and the HTC Vive, can present stimuli with precise and accurate stimulus durations.

## Experiment 2

### Introduction

Cognitive Neuroscience requires a mapping of internal neural states and cognitive processes. A prerequisite to successfully relate both is to infer a cognitive system’s current state, which can only be done from behavior. Response times and differences in reaction times are one of the most important behavioral measures available to infer cognitive processes. Hence, a reliable and precise determination of participants’ responses is essential for cognitive neuroscience since it allows inferring the mental processes to be related to neural events.

Furthermore, in order to relate functional data and ongoing cognitive processes, it is vital to accurately determine the points in time when specific sensory, cognitive, or motor events occur. For instance, studies dealing with time-sensitive functional measures, such as EEG, usually utilize the onset of a stimulus or the response time as markers to synchronize with a functional measure.

However, game engines such as UE4 are not designed for this kind of tasks and hence do not provide the necessary timing procedures allowing precise and accurate time measurements. For instance, precise reaction time measurements require that button events are registered in parallel to the stimulus presentation. UE4 registers button events only once per frame resulting in uncertainties in the range of one frame duration.

Furthermore, as described above, the actual presentation of stimuli is controlled by a VR runtime software such as SteamVR rather than by UE4 itself, which makes it impossible to accurately measure the onset time of a stimulus with UE4’s build-in timing procedures.

Therefore, the aim of Experiment 2 was to determine the precision and accuracy of time measurements with UE4 and thereby to investigate the limitations of UE4’s build-in timing procedures. Additionally, the precision and accuracy of reaction time measurements of PsychoPy and Presentation, two experimental software packages commonly used in cognitive neuroscience, were collected and compared against the results obtained with UE4.

### Material and methods

All the settings of UE4 and SteamVR in Experiment 2 were identical to those used in Experiment 1.

**Fig 3:**
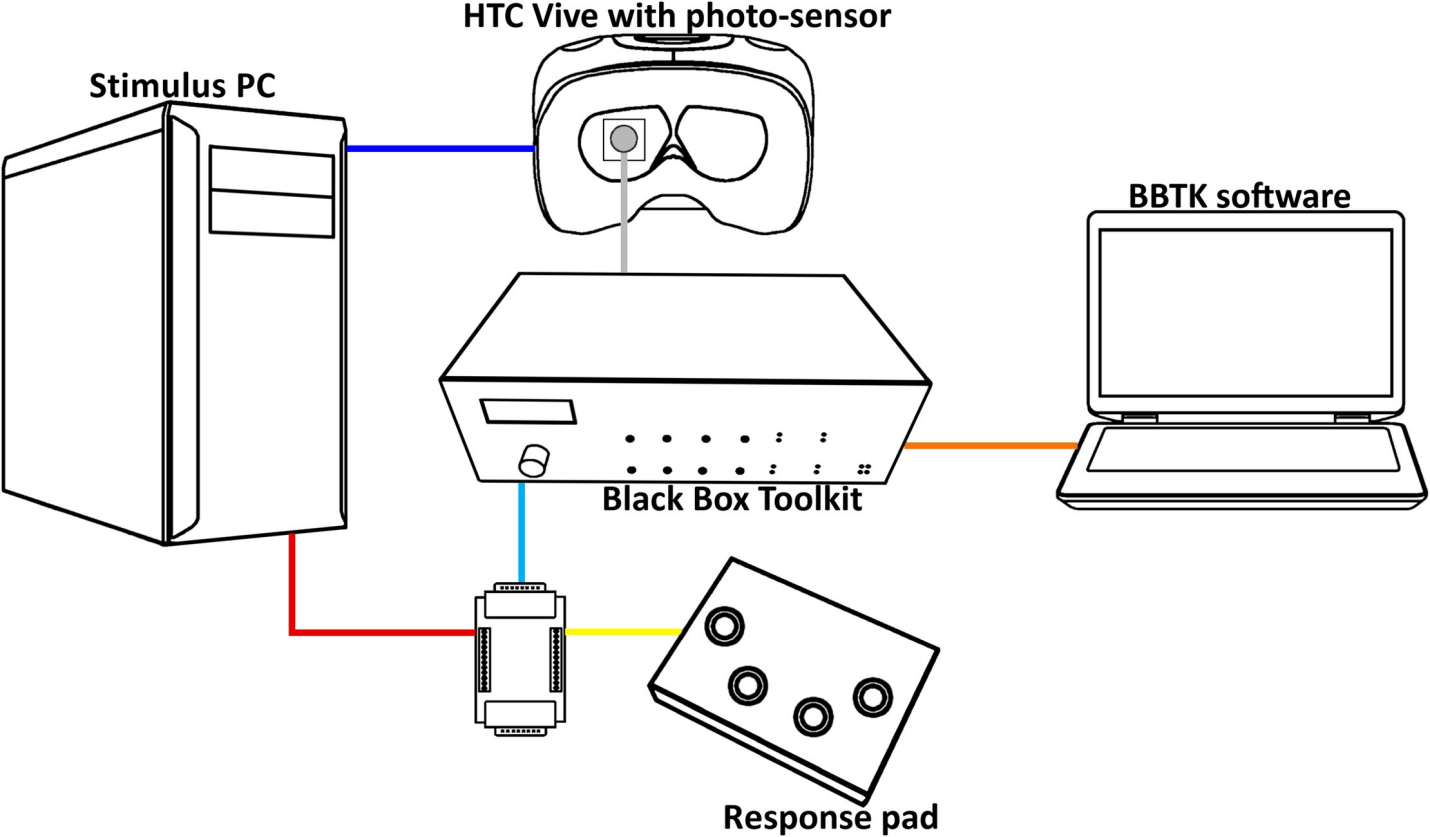
BBTK setup of the reaction time measurements.

Again, the BBTK was used to determine the precision and accuracy of time measurements, in particular, the precision of reaction time measurements. The BBTK’s photo-sensor was used to measure the onset of a stimulus on the display, and a 1000 Hz USB response device, connected to the BBTK and the stimulus computer, was set to elicit a response when the photo-sensor signaled the onset of a stimulus (Fig 3). The BBTK software allowed generating response schedules in which a sequence of response times, as well as corresponding response durations (i.e., the duration of a button press) for a number of trials, was defined. This procedure allowed the BBTK to simulate a realistic reaction time pattern. The sequence of reaction times was obtained from a previously acquired data set collected in a behavioral experiment.

We used a well-established procedure to test the precision and accuracy of reaction time measurements [20,36,41]. Again, simple black and white transitions were used as visual stimuli. However, in contrast to Experiment 1, in which the black and white transitions were restricted to a square in the middle of the display, in Experiment 2, the black and white transitions involved the entire display. The latter was done because the tests involving PsychoPy and Presentation were conducted using an LCD monitor. As previously mentioned, in contrast to the HTC Vive’s globally illuminated display, on LCD monitors the image is built up line-wise from top to bottom causing varying measurement errors depending on the position of the stimulus on the screen. Stimulus onsets were measured at the uppermost position in the left corner of the monitor to minimize hardware-related measurement errors when testing PsychoPy or Presentation. Furthermore, since LCD monitors have different and often inferior temporal properties as compared to OLEDs [42], the Samsung SyncMaster 2233 was used, which has previously been shown to provide sufficiently precise timing for vision research [43]. The tests of PsychoPy (v1.90.3) and Presentation (v20.3 02.25.19) were conducted with a refresh rate of 60 Hz.

**Fig 4:**
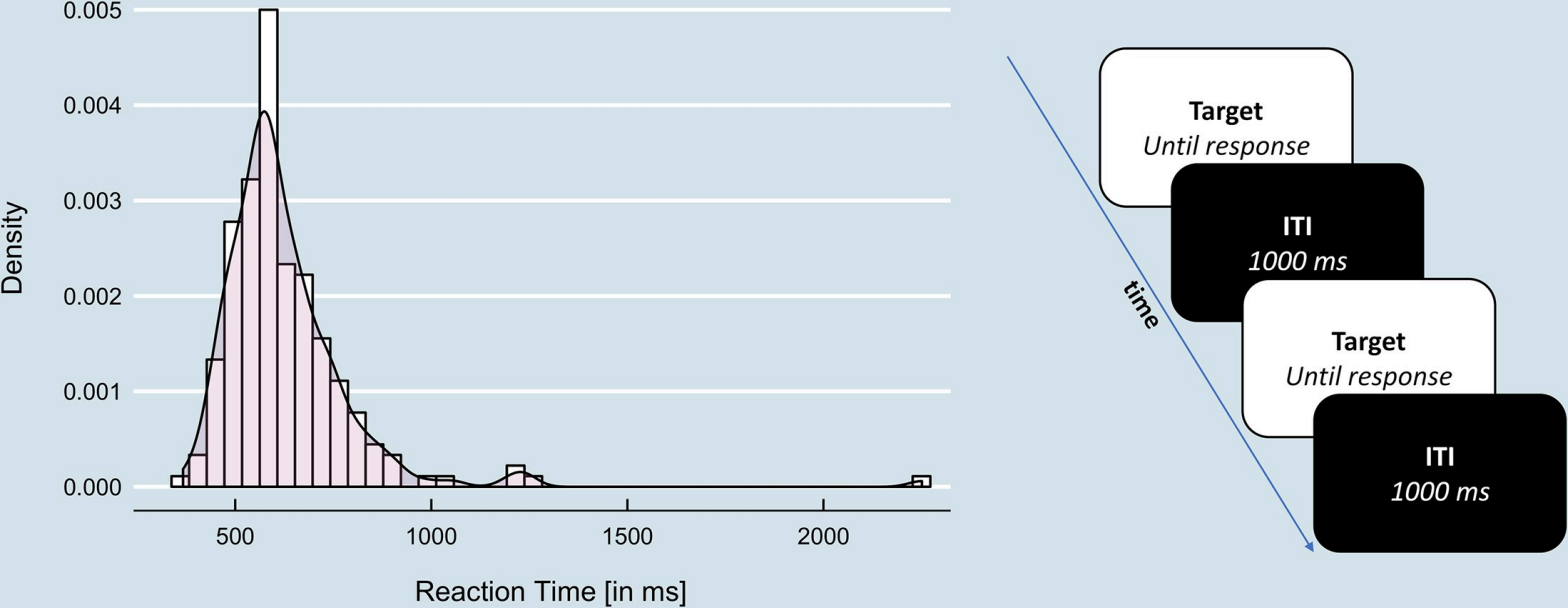
Example of one response schedule (left) and reaction time task of Experiment 2 (right).

In Experiment 2, the white stimulus was defined as the target stimulus eliciting a response by the BBTK. The target stimulus remained on the display until a response was given. Following the response, the stimulus turned black for 1 second and turned white again until the following response was given (Fig 4). This procedure allowed to compare the true reaction times as generated by the BBTK with the reaction times recorded by the stimulus software and to determine the reaction time errors as well as its variability.

All three software packages were tested in five runs of 200 trials each. For each of the five runs, a different response time schedule was applied. In order to represent realistic reaction time distributions, we assigned reaction times of human participants measured in a previously acquired dataset [44]. The reaction times of the first 200 trials of five randomly selected participants were used to generate five response time schedules (Fig 4). The response duration was kept constant and was set to 100 ms in all trials. For each software package, the same response time schedules and testing procedures were used.

All tests were conducted on a Windows 10 platform. The specification of the computer was an Intel i7-8700 CPU, 32 GB DDR4 RAM, with an Nvidia GTX 1080 graphics card.

### Results

The mean results for the three tested software packages are presented in Table 2. Both PsychoPy and Presentation showed comparable and only small overall reaction time errors that led to a mean overestimation of the reaction times of approximately 4 ms. Furthermore, the variability of the reaction time errors was comparably small in both PsychoPy and Presentation.

**Table 2:** Overview of measurement errors, standard deviation, minimum and maximum error for each software package (in ms).

Software	Mean error	SD	Min	Max
**Presentation**	3.964	0.345	3.00	5.00
**PsychoPy**	4.337	0.483	3.00	6.00
**UE4**	55.073	3.326	49.00	61.00

In contrast, UE4 showed substantial errors, with highly variable reaction time measurements with a mean measurement error of +55 ms and variability in an error range of 12 ms. Two-sample Welch t-tests were conducted to compare the mean error of UE4 with each standard software package. The comparison of UE4 (M = 55.073, SD = 3.326) and Presentation (M = 3.964, SD = 0.345) resulted in significant different mean errors t(1020) = 483.33, p < 0.05). Similarly, the comparison between UE4 and PsychoPy (M = 4.337, SD = 0.483) was significant t(1041) = 477.36, p < 0.05). Additionally, F-tests resulted in significant differences of the variance between UE4 and Presentation (F(1,999) = 93.103, p < 0.05) as well as between UE4 and PsychoPy (F(1,999) = 47.344, p < 0.05).

## Discussion

Experiment 2 aimed to quantify the precision and accuracy of reaction time measurements of UE4 and SteamVR in combination with the HTC Vive VR system. The tests illustrate the limitations of the build-in timing procedures of UE4. To obtain an estimate of the precision and accuracy provided by established software toolboxes for neuroscientific experiments in a non-VR context, we applied the same testing procedure to Presentation and PsychoPy. While both Presentation and PsychoPy provided precise and accurate reaction time measurements, reactions times measured with UE4’s build-in timing procedures were highly variable with a high mean measurement error.

The variability of the reaction time measurements can be accounted for by the fact that button events in UE4 are not registered in parallel. Instead, response events are only recorded once per frame, hence resulting in an error range of one frame duration.

In the tests reported above, stimulus onset times were determined after the command to change the stimulus color from black to white. The high overall mean error illustrates a delay between the moment the software executes the command to show a stimulus and the actual stimulus presentation time. This delay stems from several processing steps preceding the actual stimulus presentation, including a transfer of control over the stimulus presentation from UE4 to SteamVR.

However, these results were not surprising since these are known difficulties, and different frameworks have been suggested to simplify data collection for behavioral experiments [45,46] and to integrate and synchronize multiple data streams from different hardware devices, such as EEG or eye-tracking [14,16,47,48].

One framework mainly focused on the precision of reaction time measurements in combination with Unity and the HTC Vive [15]. In this study, the authors used an Arduino, a programmable microcontroller, to measure response times independently of Unity and with a sampling rate that is not limited by the refresh rate of the display. The benchmarking results illustrated that they were able to measure response times with a precision (standard deviation) of about 2.5 ms. Additionally, it was suggested that installing a photo-sensor in the HTC Vive could further improve time measurements. A small photo-sensor could measure the onset of a small peripheral stimulus appearing simultaneously with the target-stimulus. This additional stimulus could be registered directly with the Arduino.

Another recent study introduced the Unified Suite for Experiments (USE), a combination of soft- and hardware tools for the creation of experiments using Unity [14]. USE comes with a custom designed SyncBox, that is again based on an Arduino and allows to synchronize different data streams. A photo-sensor is used to measure the onset of an additional, peripheral stimulus that is presented simultaneously with the target. Benchmarking results demonstrated that the method was able to determine the stimulus onset with high precision for shorter durations. Instead, over a longer duration, the authors observed nonsystematic changes in accuracy. However, the authors provide pre-processing tools to account for these timing errors and to provide millisecond accurate stimulus onset measurements. Unfortunately, USE was not tested on HDMs directly. Instead, tests were performed on a computer monitor. Installing a photo-sensor in an HMD is technically challenging, as the photo-sensor needs to be small enough not to cause any discomfort to the participant. Nevertheless, even when small enough, the photo-sensor would be noticeable to the participant. Furthermore, this approach includes another peripheral flash stimulus that is correlated with the target. This additional stimulus could potentially confound some experiments, such as the second stimulus could distract from the target stimulus.

Therefore, in the next section, we will present a new software-based method that allows circumventing these limitations and measuring accurate and precise reaction times with UE4 and SteamVR.

## A new method for reaction time measurements with Unreal Engine and SteamVR

### Aims of the proposed method

The impact of VR on neuroscientific studies will critically depend on how easily VR experiments can be implemented and how precise control of stimulus delivery and response acquisition will be. Promising and important frameworks that aim to simplify the creation and control of VR experiments with game engines [e.g., 44, 45] are available already or are currently under development. However, so far, the progress with time-critical measurements, such as reaction times, is still insufficient and requires technically challenging setups, including additional hardware.

Here we aimed to develop a method for reaction time measurements that can provide precise and accurate measurements entirely on a software basis, which makes the need for additional hardware and complicated technical setups obsolete.

Although our method was developed mainly for UE4 and SteamVR, our aim was to ensure that the underlying principles are compatible with different game engines and the Oculus VR runtime.

However, our goal was not to develop a ready-to-use toolbox, as there are already promising toolboxes currently being developed [e.g., 45,48]. Instead, we aimed to develop and test our method as a proof-of-principle to provide a framework that can be integrated into other toolboxes.

### Method

The concept of our method is based on separating reaction time measurements from UE4 by outsourcing the measurement procedure into a background application. Hence, our method follows the same idea as the ioHub of PsychoPy. In order to provide, e.g., framerate independent response times, the ioHub monitors response events in parallel of the PsychoPy main process by running a separate process in the background [26].

In the first section, we will describe how to obtain frame rate independent measures of response times.

In the second section, we will describe how this procedure can be extended to also obtain accurate measures of stimulus onsets.

In the third section, we will provide timing benchmarks of the proposed method.

#### Measuring frame rate independent response times

**Fig 5:**
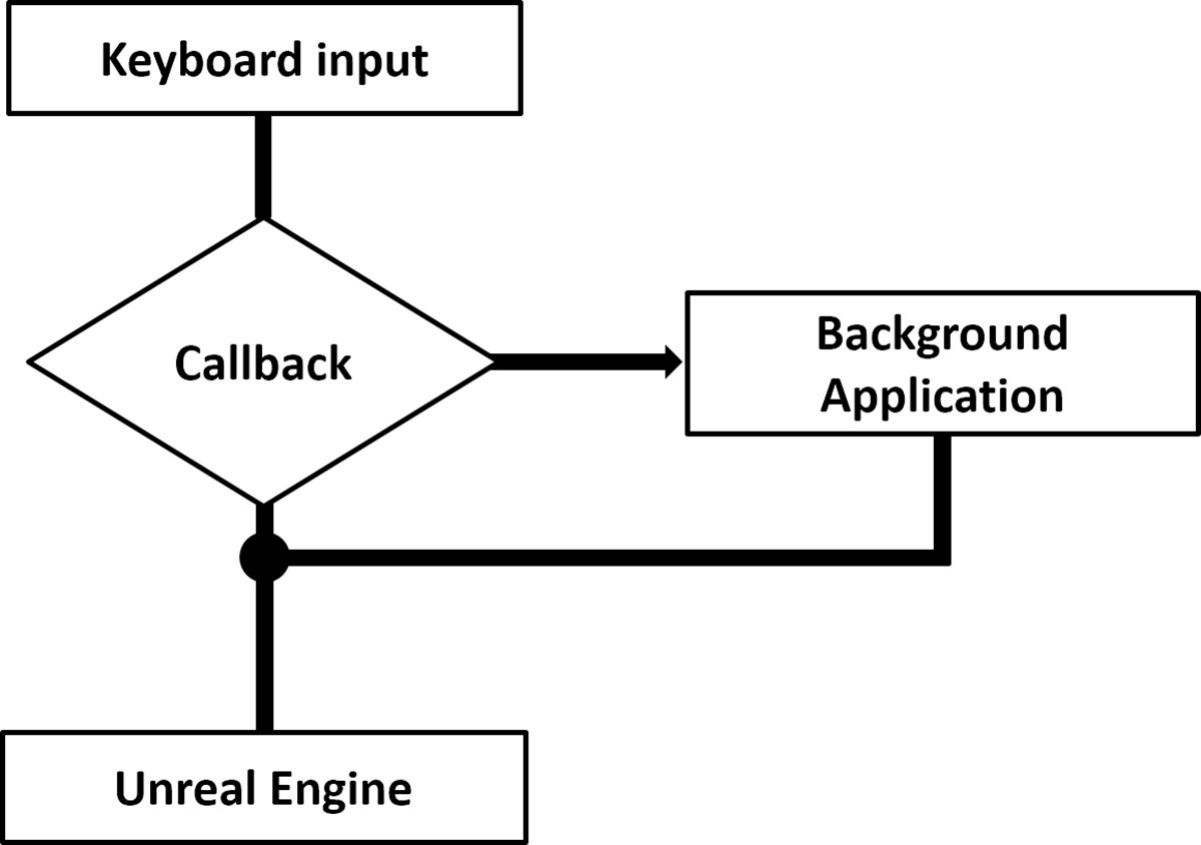
Illustration of the response time logging procedure.

For the detection of responses, a global low-level keyboard hook is used. The Microsoft Developer Network describes a hook as “a point in the system message-handling mechanism where an application can install a subroutine to monitor the message traffic in the system and process certain types of messages before they reach the target window procedure.”[49]. This procedure allows to intercept the keyboard message before Unreal receives the message and thus allows to process button input with a high sampling rate and independently of the displays frame rate (Fig 5). In particular, this procedure allowed us to increase the sampling rate of the input processing from 90 Hz to the hardware limit of the response pad of 1000 Hz.

Furthermore, to determine the reaction time, i.e., the time between stimulus onset and keyboard response, an exact determination of when a stimulus appears on the screen is necessary in addition to the exact time of a keyboard event. Therefore, it is mandatory to establish an interface that allows synchronizing our background application with UE4. For this purpose, a trigger signal (onset-trigger) is sent from UE4 to the background application, marking the time point when a stimulus is presented by using Event Objects. Event Objects can be used to exchange signals between threads or processes, indicating that a particular event has occurred [50]. As soon as the function that initiates UE4 to present the target stimulus, the onset-trigger signal is sent to our background application determining the time of a stimulus’ onset. The background application then registers the time, and the keyboard hook is activated. Once a response is given, the response time is logged, and the hook is stopped until the next onset-trigger is received from UE4.

We, therefore, ran another series of tests to assess whether this method reduces the variability of the measurement error previously observed. Again, five runs of 200 trials each, with the same response schedules used in Experiment 2 were tested.

The results demonstrated that our background application effectively reduced the variability of the response time errors down to a range of 2 ms. Conversely, the mean response time error was reduced by about half a frame duration to a mean error of 49 ms. A two-sample Welch t-test resulted in a significant difference of the mean errors between UE4 (M = 55.073, SD = 3.326) and UE4 + Hook (M = 49.030, SD = 0.381); t(1025) = 57.081, p < 0.05). A F-test comparing the variances also resulted in a significant reduced variance when measurements were conducted with the background application (F(1,999) = 76.166, p < 0.05).

The results are summarized in Table 3.

**Table 3:** Comparison of the reaction time errors, standard deviation, the minimum and maximum error of UE4 together with the hook procedure and the previous results obtained with UE4 build-in timing functions (in ms).

Software	Mean error	SD	Min	Max
**UE4**	55.073	3.326	49.00	61.00
**UE4 + Hook**	49.030	0.381	48.00	50.00

#### Measuring the stimulus onset

Accordingly, the variability of the reaction time errors can effectively be reduced. However, the mean absolute response time error of 49 ms remained relatively high compared to PsychoPy and Presentation. The remaining error indicated a delay between the moment UE4 receives the command to show a stimulus and the time when the stimulus appeared on the screen. A closer look at the rendering processes of UE4 and SteamVR revealed the origin of this delay and allowed compensating for it.

**Fig 6:**
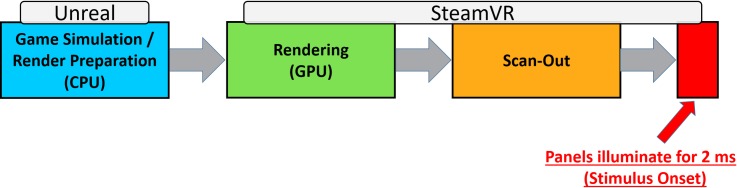
Basic illustration of the graphics pipeline.

3D real-time rendering is accomplished within a pipelined architecture called graphics pipeline. Fig 6 coarsely depicts the conceptual stages for the graphics pipeline. The first stage consists of the game simulation and rendering preparation. Before the scene can be rendered, the system needs to determine the locations of objects to be rendered in the scene. The game simulation accomplishes this task. The game simulation calculates all the logic and transforms, i.e., the position, orientation, and scale of all objects in the scene, taking into account inputs by the user, animations, physics simulation, and AI. The rendering preparation then determines what is currently visible in the scene and creates a list of all the objects that need to be rendered and passes this list to the GPU. These steps are mostly processed on the CPU and controlled by UE4. The next stage is the actual geometry rendering, which is processed on the GPU. The final stage before the frame gets visible on the displays is referred to as scan-out. Scan-out describes the transfer of the image via HDMI, thereby loading it onto the displays. After scan-out completion, the display panels illuminate, and the frame gets visible. The last two stages are controlled by SteamVR rather than UE4.

Calling the function to present a target stimulus in UE4 is just the first step of a whole cascade of processes required to display a stimulus. Timestamping a stimulus event at the beginning of this cascade (when the function to present the stimulus is called) is far too early, resulting in the measurement errors observed in Experiment 2.

However, the graphics pipeline illustrates that it is not possible to measure the stimulus onset directly via UE4. After submitting the rendering commands to the GPU, SteamVR determines when the frame is sent to the HMD. This is further complicated by the fact, that unlike in rendering to a normal computer monitor, the scan-out has to be finished before the displays light up and the stimulus is presented.

A more detailed illustration and description of the temporal characteristics of the graphics pipeline is shown in the supporting information (S2 Fig and S1 Text).

#### Prediction

The method that allows determining the true stimulus onset time is based on prediction. In particular, in order to predict the exact stimulus onset, a procedure can be implemented that is based on SteamVR’s procedures to predict the user’s pose. One of the critical determinants to provide a compelling VR experience is to reduce the so-called Motion-To-Photon latency. Motion-To-Photon latency describes the time that is required for a user’s head movement to be fully reflected in the HMD. As described above, the system has to determine the layout of the scene before the scene can be rendered. One of the critical factors that determine what is currently visible and what hence needs to be rendered is the user’s pose, i.e., the position and the orientation of the HMD. However, rendering takes time and induces undesired latencies, thereby reducing the quality of the VR experience. Furthermore, latency is one of the main drivers of motion sickness, a side effect that leads to symptoms like nausea, dizziness, or vertigo [51].

SteamVR’s strategy to reduce the Motion-To-Photon latency is to predict the user’s pose, giving the best estimate for the users’s pose when the frame is displayed on the headset, to compensate for the latency in the system. To obtain an accurate prediction, the system needs to estimate when the currently rendered frame will finally be displayed. All necessary functions to predict when a frame will be displayed are provided within SteamVR’s application programming interface (API) OpenVR. The proposed method is synchronized to SteamVR’s pose prediction processes and further utilizes the provided functions to estimate the exact onset time of a frame.

SteamVR allows predicting when a frame will be presented. The prediction is purely temporal and does not contain any information on the frame’s content. In other words, it holds information on when a frame will be presented but not on what will be presented in that frame. Trial specific information is only available through UE4. Hence, in order to be able to predict the onset time of a specific event such as the stimulus onset, it is necessary to combine information about the contents and temporal orders of trials from UE4 and the exact frame-timing from SteamVR. Our background application can meet this challenge.

**Fig 7:**
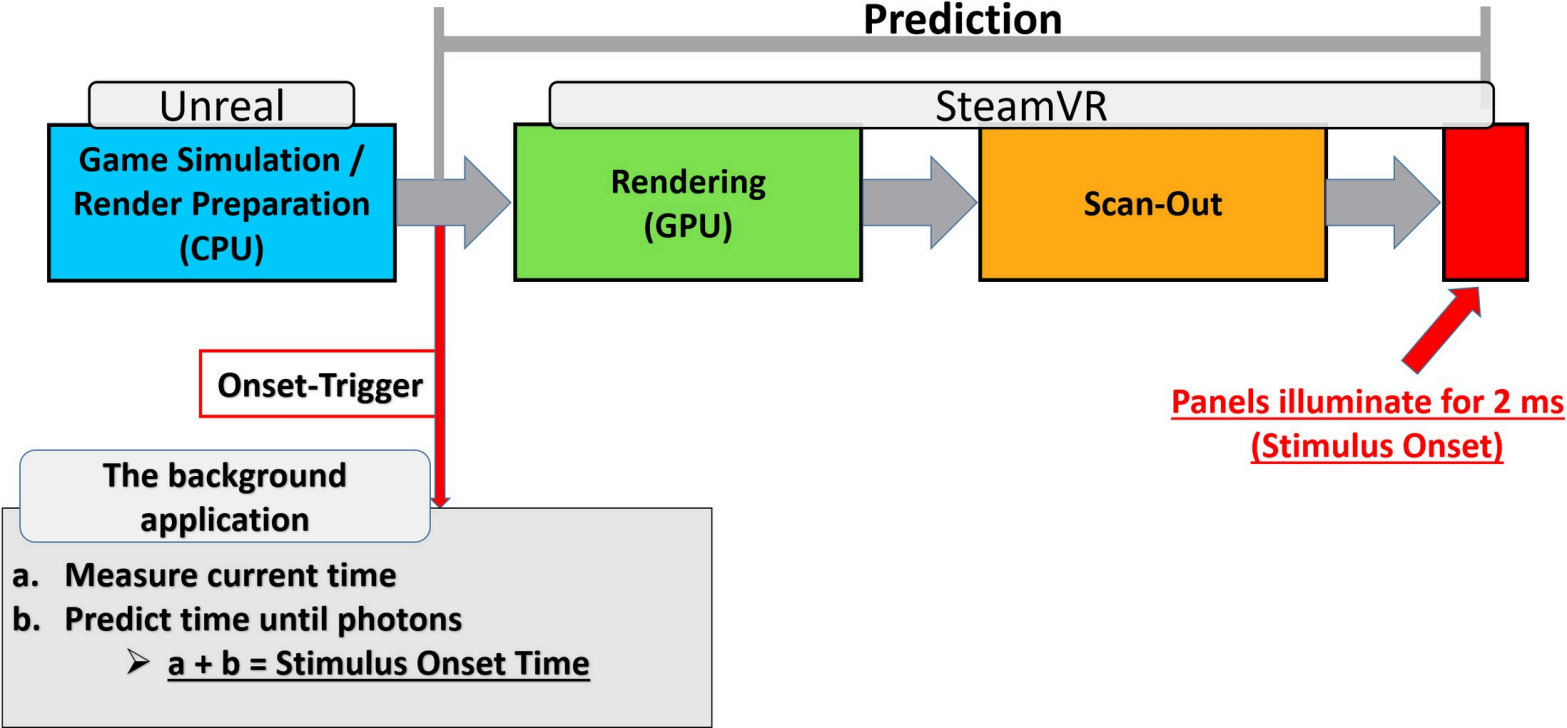
Illustration of the stimulus onset prediction.

In particular, our background application first synchronizes with the trial-timing of UE4. For this synchronization, UE4 sends a trigger-signal (onset-trigger) to the background application whenever a frame marking the stimulus-onset is about to be submitted to the GPU, right before the control over the rendering is handed over to SteamVR. Following the trigger-signal, the background application stores a timestamp and fetches the necessary information about the frame-timing from SteamVR, which is then used to predict the stimulus-onset (Fig 7).

A more detailed description of the processing steps involved in the prediction can be found in the supporting information, S3 Fig and S2 Text.

#### Assumptions

Onset prediction is based on a few assumptions that have to be met. The first assumption is that there are no additional frame buffers in the graphics pipeline. Moreover, the Experiment must run at a constant frame rate without dropped or reprojected frames and without the use of Motion Smoothing. We recommend turning off Motion Smoothing completely. However, Asynchronous Reprojection cannot be turned off, and to prevent frame drops and reprojected frames, optimization of the experiment is mandatory. Yet, even with such optimization, occasional performance problems cannot be ruled out entirely, and, hence, should be controlled for. Therefore, a control mechanism was implemented to detect trials affected by dropped or reprojected frames. The background application stores the number of dropped or reprojected frames of every experimental trial and stores it into a log file. Whenever a trial contains dropped or reprojected frames, the trial should be excluded from the analysis, since the validity of the measured reaction time cannot be guaranteed.

### Validation of the method

To ensure that the above-described method can measure precisely and accurately reaction times, we repeated the testing procedure of Experiment 2. The three conditions of Experiment 1 were tested to determine the precision and accuracy of the method. Furthermore, the tests were conducted on both computers, previously used in Experiment 1, to determine the reliability of the method on different computer hardware. Overall, the method was tested in 6000 trials.

### Results

The mean results for the three conditions are presented in Table 4. The mean measurement error was 1.44 ms within a range of 2 ms across conditions and computers. Performance remained good during all tests, as no dropped or reprojected frames were observed.

To test for differences in precision or accuracy between conditions or computers, a two factorial ANOVA with the factors *Condition* [Simple (M = 1.446 ms, SD = 0.498), Complex-Static (M = 1.443, SD = 0.497), Complex-Moving (M = 1.439, SD = 0.496)] and *Computer* (Computer 1, Computer 2) was performed. The ANOVA revealed no significant main effects—neither for the factor *Condition* (F(1,2) = 0.102, p = 0.903), nor for the factor Computer [Computer 1 (M = 1.450 ms, SD = 0.4975) and Computer 2 (M = 1.435, SD = 0.4965); (F(1,1) = 1.305, p = 0.253)]. The interaction between the factors *Condition* and *Computer* was also not significant (F(1,2) = 0.345, p = 0.709). The individual results of both computers separately can be found in the supporting information (see S8 Table and S9 Table).

**Table 4:** Overview of mean reaction time errors, standard deviation, minimum and maximum error for each condition (in ms).

Condition	Mean error	SD	Min	Max
**Simple**	1.446	0.498	1.00	3.00
**Complex-Static**	1.443	0.497	1.00	2.00
**Complex-Moving**	1.439	0.496	1.00	2.00
**Overall**	1.442	0.497	1.00	3.00

## Discussion

In Experiment 2, we observed highly variable reaction time measurements with inaccurate stimulus onset measurements with Unreal Engine, SteamVR, and the HTC Vive VR system, when using the built-in timing procedures.

Hence, we developed a new software-method to provide precise and accurate reaction time measurements with Unreal Engine and SteamVR. Instead of measuring reaction times in Unreal Engine directly, the measurement part was outsourced to a background application. Timing benchmarks indicate that the method allows precise and accurate reaction time measurements.

We made the source code of the background application and the project files as well as project builds of the UE4 projects as tested in this experiment available on https://jugit.fz-juelich.de/inm3/timingvr. The commits to the UE4 source code are available on https://github.com/INM3FZJ/UnrealEngine/tree/TimingVR. However, note that the background application was developed as a proof-of-principle rather than a ready-to-use toolbox, as the current implementation is limited in its functionality and was custom-made to fit the particular requirements of the current research project.

## General discussion

The study aimed to investigate the precision and accuracy in stimulus presentation durations and reaction time measurements with Unreal Engine and SteamVR in combination with the HTC Vive VR system.

In Experiment 1, we tested the precision and accuracy of stimulus presentations to determine potential discrepancies between the stimulus timing defined by the researcher and the actual stimulus timing on the display. Our measurements revealed that the precise frame rate of the HTC Vive slightly differs from the information given in the HTC Vive manual. The HMD’s empirical frame rate was determined as 89.53 FPS rather than the indicated 90 FPS. Importantly, the frame rate remained stable across conditions and in combination with different computer hardware. The same frame rate was confirmed in a second HTC Vive system, suggesting that the observed frame rate was device specific.

The results of Experiment 1 indicate that the stimulus timing was precise and accurate and remained stable across different computer hardware configurations as well as across different rendering workload requirements. UE4 in combination with SteamVR and the HTC Vive VR system, therefore, appears to be suitable for time-critical visual stimulus presentations. The so-called global-onset displays as used in the HTC Vive come with another significant advantage. Due to the nature of how the image is updated in LCD and CRT monitors, the measurement error of the stimulus onset is correlated with the position of the stimulus on the display. In contrast, the displays of the HTC Vive load the entire image before the displays illuminate. Consequently, when used in combination with precise time measurements, the global-onset displays allow determining the onset of a stimulus without variable measurement errors due to the stimulus position.

Experiment 2 aimed to investigate the limitations of the build-in timing procedures of UE4 for reaction time measurements. Timing benchmarks resulted in highly variable measurement errors more than ten times higher as those obtained with Presentation and PsychoPy.

Hence, a new software-based method as a proof of principle was developed that allows precise and accurate reaction time measurements with UE4 in combination with SteamVR. The new method measures reaction times as independent as possible from the procedures implemented in UE4. This is achieved by outsourcing the measurement procedure into a background application that allows circumventing the limitations of UE4’s build-in timing procedures. In a first step, a subroutine implemented into the background application monitors and processes keyboard messages before they reach UE4 and hence allows the collection of frame rate independent response times. In a second step, a prediction algorithm, based on SteamVR’s pose prediction procedures, was implemented to determine the true stimulus onset accurately. Timing benchmarks show that the method accurately and precisely determines stimulus onsets and hence in combination with veridical response time acquisition allows validly measuring reaction times.

The method presented here could help to simplify experimental procedures, as is allows to measure stimulus events and response times without a need for complex hardware and sensor setups.

Please note, the background application introduced here, is a proof-of-principle rather than a ready-to-use toolbox. Our aim was to provide an example of a framework for reaction time measurement that could be integrated into other toolboxes.

Although our method was tested with UE4, the underlying principles are, at least in principle, also applicable to other game engines such as Unity, as it is only necessary to provide a trigger signal to synchronize the reaction time measurements of the background application with the trial-timing of the game engine. Furthermore, to our understanding, the principles to predict the stimulus-onset should also apply to the Oculus VR runtime software. Oculus uses a similar mechanism to predict the user’s pose as SteamVR, and the Oculus API also provides the tools necessary to predict the onset time of a frame and hence could be used to predict the stimulus onset. Additionally, the version of the background application here presented was already designed to also support different HMD’s other than the HTC Vive.

Furthermore, the ability to accurately and precisely determine the time of stimulus events, such as stimulus onset, and response times, is not only essential for reaction time measurements. Instead, a precise recording of events within an experiment is mandatory for the analysis of all time-sensitive functional measures, such as EEG or MEG. Therefore, the method could also be extended, that stimulus and response events could be used as markers to synchronize with time-sensitive functional measures.

However, it should be noted that the method heavily relies on game engines and VR runtime software (here UE4 and SteamVR). Therefore, a limitation of this method is that future updates of these third-party tools might cause compatibility problems or result in incorrect measurements. Hence, it will be important to regularly validate the precision and accuracy of time measurements with new software releases.

Furthermore, we developed and tested the method only for standard keyboard input and did not include support for the HTC Vive’s motion controllers. Motions controllers are increasingly used to investigate naturalistic behavior, as they allow to track the hand movements of the participant [e.g., 52,53]. Future research needs to assess timing errors related to motion controllers.

In sum, the HTC Vive, in combination with UE4 and our newly developed tool, constitutes a precise and valuable instrument for neuroscientific research and visual sciences. Besides its excellent performance, regarding accuracy and precision it allows taking advantage of all the benefits that VR offers. When used in combination with precise research tools, VR has the potential to implement new paradigms involving large fields of view of realistic and three-dimensional environments. It allows integrating a variety of behavioral responses, increasing the ecological validity of neuroscientific experiments, which may lead to more generalizable and valid explanations regarding cognitive processes.

## Supporting information

S1 File (DOCX)

S1 Fig Screenshot of UE4’s realistic rendering sample. (TIF)

S2 Fig Illustration of the processing stages that a frame has to pass before it is presented to the display panels. (TIF)

S3 Fig Framework for the stimulus onset prediction. (TIF)

S1 Table Results across all conditions of Computer 1 (in ms). (DOCX)

S2 Table Results across all conditions of Computer 2 (in ms). (DOCX)

S3 Table Results of the simple condition across computers (in ms). (DOCX)

S4 Table Results of the Complex-Static condition across computers (in ms). (DOCX)

S5 Table Results of the Complex-moving condition across computers (in ms). (DOCX)

S6 Table Results of the simple condition of HMD 1 (in ms). (DOCX)

S7 Table Results of the simple condition of HMD 2 (in ms). (DOCX)

S8 Table Overview of mean reaction time errors, standard deviation, minimum and maximum error for each condition of Computer 1 (in ms). (DOCX)

S9 Table Overview of mean reaction time errors, standard deviation, minimum and maximum error for each condition of Computer 2 (in ms). (DOCX)

S1 Text Temporal characteristics of the graphics pipeline with UE4 and SteamVR. (DOCX)

S2 Text Stimulus onset prediction. (DOCX)
