# Supplementary material for: Accuracy and precision of stimulus timing and reaction times with Unreal Engine and SteamVR
Source: PLoS One. 2020 Apr 8;15(4):e0231152. doi: 10.1371/journal.pone.0231152 (PMC7141612; doi:10.1371/journal.pone.0231152)
Supplement: S1 File — (DOCX) [file pone.0231152.s001.docx]

*------- Insert Fig S1 about here --------*

**Fig S1. Screenshot of UE4’s Realistic Rendering sample.**

The screenshot shows the original environment without the modifications as used in this study.

*------- Insert Fig S2 about here --------*

**Fig S2. Illustration of the processing stages that a frame has to pass before it is presented to the display panels.**

*------- Insert Fig S3 about here --------*

**Fig. S3. Framework for the stimulus onset prediction.**
