# Supplementary figures and images for: Accuracy and precision of stimulus timing and reaction times with Unreal Engine and SteamVR
Source: PLoS One. 2020 Apr 8;15(4):e0231152. doi: 10.1371/journal.pone.0231152 (PMC7141612; doi:10.1371/journal.pone.0231152)

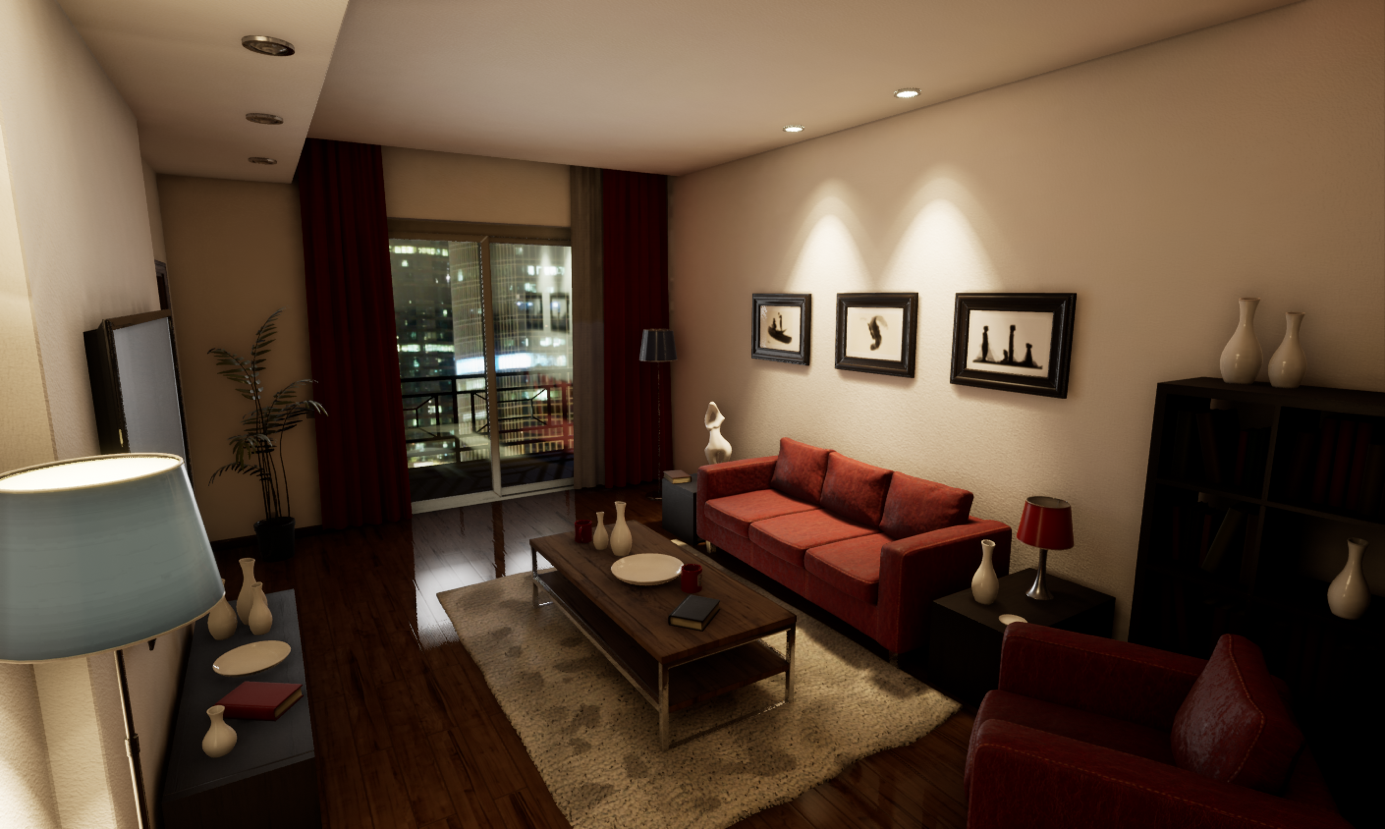

Supplement: S1 Fig — (TIF) [file pone.0231152.s002.tif]

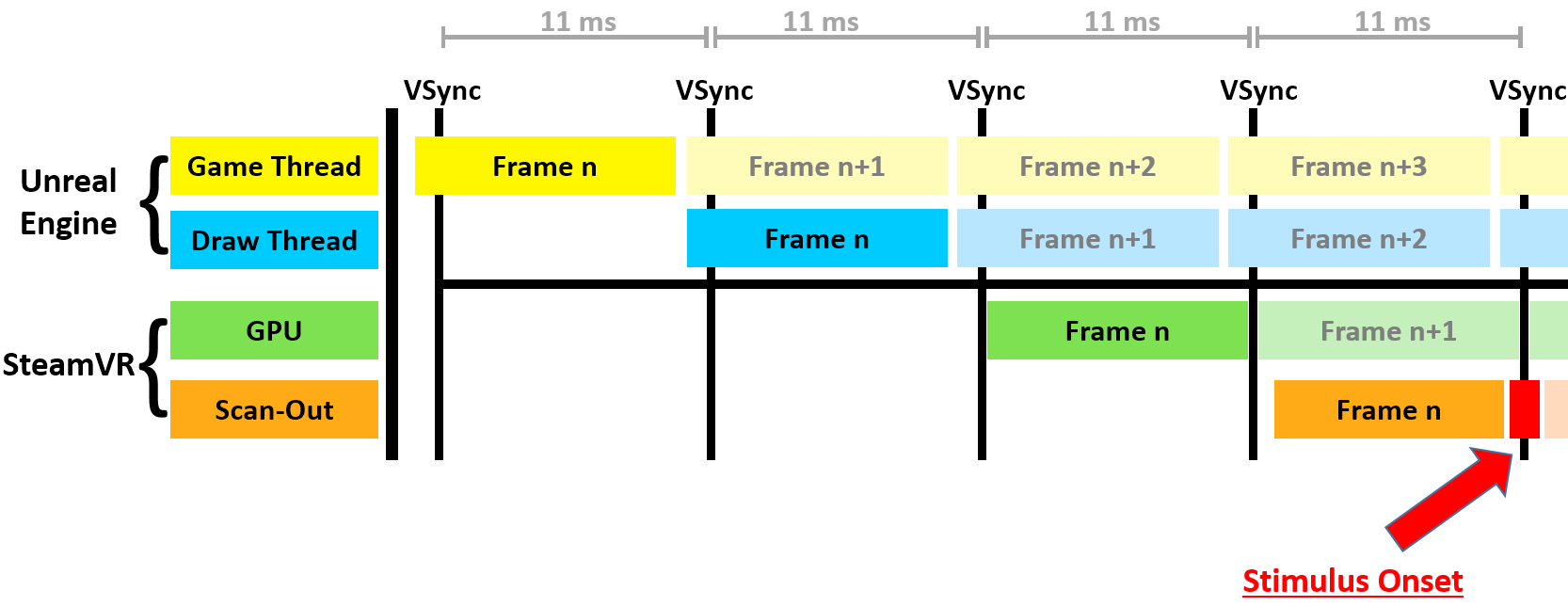

Supplement: S2 Fig — (TIF) [file pone.0231152.s003.tif]

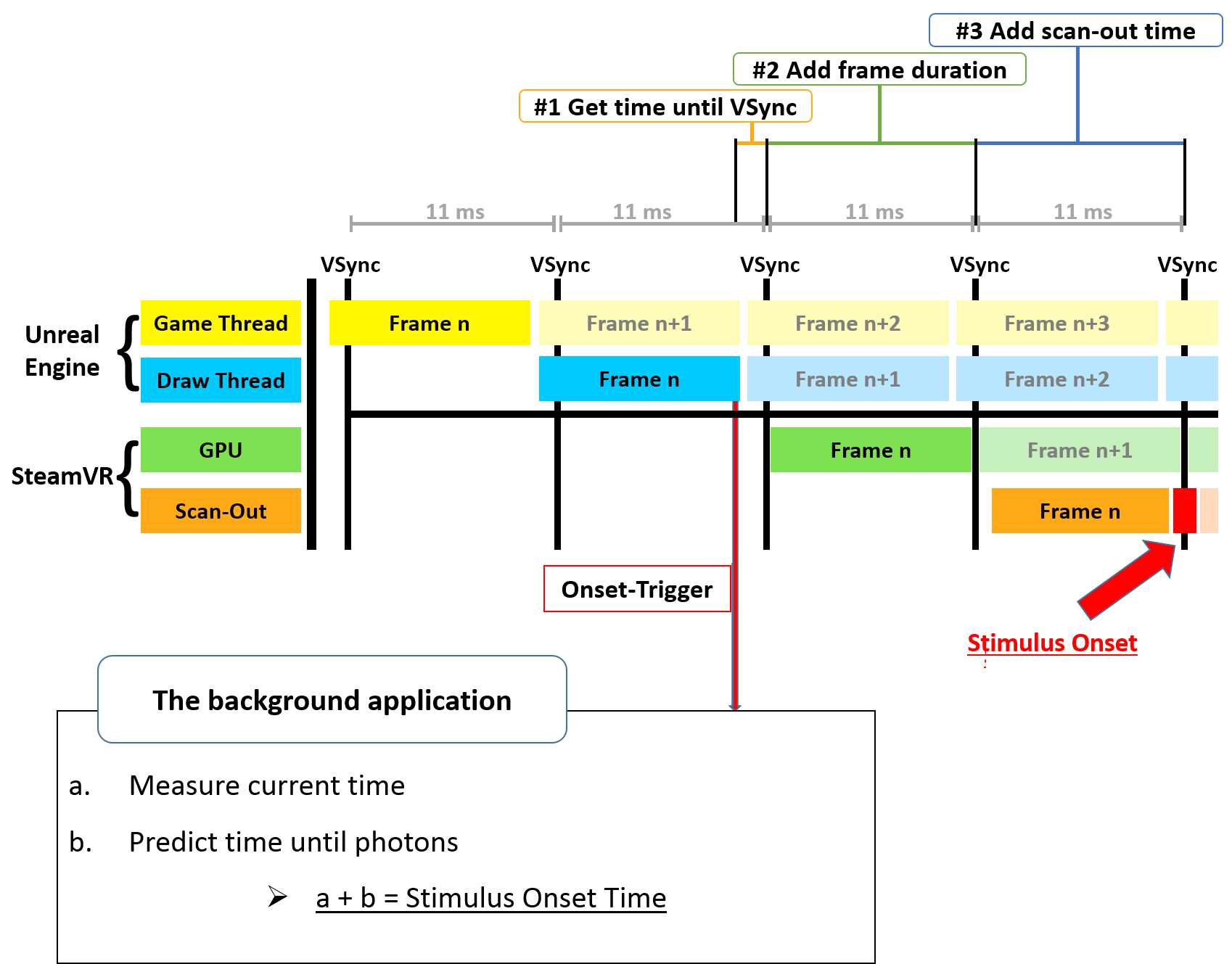

Supplement: S3 Fig — (TIF) [file pone.0231152.s004.tif]
