# Supplementary material for: Accuracy and precision of stimulus timing and reaction times with Unreal Engine and SteamVR
Source: PLoS One. 2020 Apr 8;15(4):e0231152. doi: 10.1371/journal.pone.0231152 (PMC7141612; doi:10.1371/journal.pone.0231152)
Supplement: S2 Table — (DOCX) [file pone.0231152.s006.docx]

**S2 Table. Results across all conditions of Computer 2 (in ms).**

| **expected duration** | **mean** | **sd** | **min** | **max** | **mean duration white** |
| --- | --- | --- | --- | --- | --- |
| **2000** | 2010.59 | 0.121 | 2010.50 | 2010.75 | 996.06 |
| **1000** | 1005.30 | 0.098 | 1005.25 | 1005.50 | 493.41 |
| **400** | 402.12 | 0.125 | 402.00 | 402.25 | 191.82 |
| **200** | 201.06 | 0.106 | 201.00 | 201.25 | 91.29 |
| **133.33** | 134.04 | 0.091 | 134.00 | 134.25 | 57.80 |
| **66.66** | 67.02 | 0.068 | 67.00 | 67.25 | 24.27 |
| **22.22** | 22.34 | 0.120 | 22.25 | 22.50 | 1.93 |

*The first column represents the expected duration for the black and white stimulus cycles. The last column represents the measured durations of the white stimulus (in ms).*
