# Supplementary material for: Accuracy and precision of stimulus timing and reaction times with Unreal Engine and SteamVR
Source: PLoS One. 2020 Apr 8;15(4):e0231152. doi: 10.1371/journal.pone.0231152 (PMC7141612; doi:10.1371/journal.pone.0231152)
Supplement: S4 Table — (DOCX) [file pone.0231152.s008.docx]

**S4 Table. Results of the Complex-Static condition across computers (in ms).**

| **expected duration** | **mean** | **sd** | **min** | **max** | **mean duration white** |
| --- | --- | --- | --- | --- | --- |
| **2000** | 2010.58 | 0.116 | 2010.50 | 2010.75 | 996.10 |
| **1000** | 1005.29 | 0.090 | 1005.25 | 1005.50 | 493.41 |
| **400** | 402.12 | 0.125 | 402.00 | 402.25 | 191.82 |
| **200** | 201.06 | 0.105 | 201.00 | 201.25 | 91.30 |
| **133.33** | 134.04 | 0.090 | 134.00 | 134.25 | 57.79 |
| **66.66** | 67.02 | 0.067 | 67.00 | 67.25 | 24.28 |
| **22.22** | 22.34 | 0.120 | 22.25 | 22.50 | 1.93 |

The first column represents the expected duration for the black and white stimulus cycles. The last column represents the measured durations of the white stimulus (in ms).
