# Supplementary material for: Accuracy and precision of stimulus timing and reaction times with Unreal Engine and SteamVR
Source: PLoS One. 2020 Apr 8;15(4):e0231152. doi: 10.1371/journal.pone.0231152 (PMC7141612; doi:10.1371/journal.pone.0231152)
Supplement: S7 Table — (DOCX) [file pone.0231152.s011.docx]

**S7 Table. Results of the Simple condition of HMD 2 (in ms).**

| **expected duration** | **mean** | **sd** | **min** | **max** | **mean duration white** |
| --- | --- | --- | --- | --- | --- |
| **2000** | 2010.56 | 0.107 | 2010.50 | 2010.75 | 996.03 |
| **1000** | 1005.28 | 0.081 | 1005.25 | 1005.50 | 493.39 |
| **400** | 402.11 | 0.124 | 402.00 | 402.25 | 191.81 |
| **200** | 201.06 | 0.104 | 201.00 | 201.25 | 91.28 |
| **133.33** | 134.04 | 0.089 | 134.00 | 134.25 | 57.77 |
| **66.66** | 67.02 | 0.066 | 67.00 | 67.25 | 24.26 |
| **22.22** | 22.34 | 0.120 | 22.25 | 22.50 | 1.92 |

The first column represents the expected duration for the black and white stimulus cycles. The last column represents the measured durations of the white stimulus.
