# Supplementary material for: Accuracy and precision of stimulus timing and reaction times with Unreal Engine and SteamVR
Source: PLoS One. 2020 Apr 8;15(4):e0231152. doi: 10.1371/journal.pone.0231152 (PMC7141612; doi:10.1371/journal.pone.0231152)
Supplement: S8 Table — (DOCX) [file pone.0231152.s012.docx]

**S8 Table. Overview of mean reaction time errors, standard deviation, minimum and maximum error for each condition of Computer 1 (in ms).**

| **Condition** | **Mean error** | **SD** | **Min** | **Max** |
| --- | --- | --- | --- | --- |
| **Simple** | 1.446 | 0.4973 | 1.00 | 2.00 |
| **Complex-Static** | 1.451 | 0.4978 | 1.00 | 2.00 |
| **Complex-Moving** | 1.452 | 0.4979 | 1.00 | 2.00 |
| **Overall** | 1.446 | 0.4973 | 1.00 | 2.00 |
