# Supplementary material for: Accuracy and precision of stimulus timing and reaction times with Unreal Engine and SteamVR
Source: PLoS One. 2020 Apr 8;15(4):e0231152. doi: 10.1371/journal.pone.0231152 (PMC7141612; doi:10.1371/journal.pone.0231152)
Supplement: S9 Table — (DOCX) [file pone.0231152.s013.docx]

**S9 Table. Overview of mean reaction time errors, standard deviation, minimum and maximum error for each condition of Computer 2 (in ms).**

| **Condition** | **Mean error** | **SD** | **Min** | **Max** |
| --- | --- | --- | --- | --- |
| **Simple** | 1.445 | 0.4992 | 1.00 | 3.00 |
| **Complex-Static** | 1.435 | 0.4960 | 1.00 | 2.00 |
| **Complex-Moving** | 1.425 | 0.4946 | 1.00 | 2.00 |
| **Overall** | 1.445 | 0.4992 | 1.00 | 3.00 |
