# Supplementary material for: Accuracy and precision of stimulus timing and reaction times with Unreal Engine and SteamVR
Source: PLoS One. 2020 Apr 8;15(4):e0231152. doi: 10.1371/journal.pone.0231152 (PMC7141612; doi:10.1371/journal.pone.0231152)
Supplement: S1 Text — (DOCX) [file pone.0231152.s014.docx]

**S1 Text. Temporal characteristics of the graphics pipeline with UE4 and SteamVR**

Figure S2 illustrates the different stages that a frame (devoted as frame n) must pass before it is finally presented on display. The following description will focus on the temporal aspects rather than on the functional aspects of the described stages.

Please note, that every stage in the pipeline is synchronized to the vertical sync (VSync) event. Frame n is initially processed in the Game Thread where game simulation takes place. The Game Thread constitutes a relatively early stage in this processing pipeline and represents the stage where the experimental routine is executed, e.g., where the command to present the target stimulus is completed. The timestamp marking a stimulus’ onset as measured in Experiment 2 was determined at this level. After one frame duration, everything is transferred to the Draw Thread for rendering preparation. It also requires an additional full frame duration until the resulting rendering commands are passed to the GPU. The GPU then again requires another full frame duration before, finally, the frame can be scanned out to the displays. The scan-out occurs while the display is black, which requires about 9 ms until the displays light up, and the new frame is presented for about 2 ms. The last two stages are controlled by SteamVR rather than UE4, illustrated by the horizontal black line in Fig S2.

Note that both the Game Thread and the Draw Thread are shifted relative to the VSync events. This is a novelty of rendering with SteamVR. In a non-VR graphics pipeline, both the game and the draw thread would start right after a VSync event. The rendering commands would then be submitted to the GPU right after the VSync event, and everything would be buffered for one or more frame durations before the GPU would render the frame. In order to reduce latency, VR rendering occurs without frame buffering. However, submitting a frame to the GPU is time-consuming, and without frame buffering, a so-called “GPU bubble” of up to two milliseconds would be produced, in which the GPU is idle until rendering can be initiated, thus effectively reducing the total time available for rendering. The rendering commands of the Draw Thread are submitted to the GPU before the VSync event to ensure that the GPU has a full frame duration for rendering. This process is called “running start”. To ensure that both the Game Thread and the Draw Thread still have the budget of a full frame duration, with SteamVR, the calculations of both threads start a few milliseconds before the VSync event.

The scan-out is the final stage before the frame is displayed. After loading the entire display, the pixels illuminate, and the frame gets visible for about 2 ms.

When considering the above-presented graphics pipeline involved in presenting a single frame, it becomes evident that calling the function to present a target stimulus in the Game Thread is just the first step of a whole cascade of processes required to display a stimulus. Timestamping a stimulus event at the beginning of this cascade (when the function to present the stimulus is called) is far too early, resulting in the measurement errors observed in Experiment 2. However, the graphics pipeline also illustrates that it is not possible to measure the stimulus onset directly via UE4. After submitting the rendering commands to the GPU, SteamVR determines when the frame is sent to the HMD. This is further complicated by the fact, that unlike in rendering to a normal computer monitor, the scan-out has to be finished before the displays light up and the stimulus is presented.
