# Supplementary material for: Accuracy and precision of stimulus timing and reaction times with Unreal Engine and SteamVR
Source: PLoS One. 2020 Apr 8;15(4):e0231152. doi: 10.1371/journal.pone.0231152 (PMC7141612; doi:10.1371/journal.pone.0231152)
Supplement: S2 Text — (DOCX) [file pone.0231152.s015.docx]

**S2 Text. Stimulus onset prediction**

The prediction is based on the fact, that all rendering steps for VR are synchronized to the VSync events, which allows us to use VSync events as reliable time markers when a screen refresh has finished.

An exact estimate is based on accurate knowledge when (i.e., during which frame interval) the prediction was started. This can be accomplished by sending the onset-trigger from UE4 to the background application later within the processing sequence of UE4 than in the previous tests. The onset-trigger is then sent to the background application from UE4’s SteamVR plugin at the end of Draw Thread’s processing a few milliseconds before the next VSync event. This is accomplished by sending the onset-trigger signal right after a function called WaitGetPoses() returns. The WaitGetPoses() function is responsible for pose prediction and is called by UE4 after the Draw Thread has finished processing. It blocks the Draw Thread until a few milliseconds before the VSync event and then returns the predicted poses to be used for the rendering. By sending the onset-trigger right after WaitGetPoses() returns, a synchronization point with VSync events is generated and ensures that the prediction of a stimulus onset always starts in the same stage of the graphics pipeline.

The current implementation of the synchronization of UE4 and the background application works as follows. The command to show a stimulus on display is called in the Game Thread. In order to generate a precise prediction when the stimulus appears on display, one has to determine the time point when the Draw Thread in UE4 hands over stimulus processing to SteamVR. Therefore, the current background application uses two different trigger signals, one marking the moment when the command to show a stimulus is called and later a second trigger signal marking the moment when the process is handed over to SteamVR. The latter is about the exact timing and for the synchronization with the frame intervals; the former is relevant since it indicates an upcoming visual event that is important for the experimental procedure. Both signals are relevant and need to be considered unison, which creates a need to integrate or relate both at some stage. This could be done by implementing a complicated direct exchange of timing signals between the Game Thread and the Draw Thread. Alternatively, this problem could be solved by using an external interface that registers and relates both trigger signals such as our background application. The latter approach requires only minimal changes in the UE4 source code and hence avoids changes that might affect the normal working of UE4. Therefore, we decided to use this option to develop a proof-of-principle method for precise timing measures in UE4. It involves sending two trigger signals to the background application. In Game Thread, a trigger, which indicates a relevant stimulus to be presented, is sent whenever the command to show the stimulus is called. The trigger signal marking the moment when the signal is handed over from the Draw Thread to SteamVR is sent on every frame. Hence, the background application can integrate both signals in the following way. On every frame the background application receives a signal from the Draw Thread, thereby providing an exact measure on when information is passed to SteamVR, this information is only valuable when it needs to be related to a relevant visual stimulus, i.e., when it is preceded by a trigger signal indicating an upcoming visual event. Otherwise, this information can be ignored. Accordingly, our background application waits for a start-trigger from UE4, indicating an upcoming stimulus onset, and only then further processes the signal indicating the hand-over to SteamVR. When this signal is received, it takes the current time and starts predicting the actual stimulus onset. Adding the measured time to the prediction then estimates stimulus onset. In the next step, the keyboard hook is started, and the application waits until it receives a keyboard message and takes the response time. After writing the timing information into a log file, it starts again to wait for the next start-trigger.

As soon as the background application received the onset-trigger, it takes the current time and then starts predicting the time remaining until the target will be displayed.

The first step in this calculation is to determine the remaining time of the current frame interval accurately. This is achieved by calling a function that returns the time since the last VSync event. The difference between a frame duration and the time since the last VSync determines the remaining time of the frame interval. Instead of hardcoding the frame duration, we calculate the frame duration based on the HMD’s refresh rate, which is retrieved from SteamVR. This allows using the background application with different HMD and different refresh rates.

In the next step, one frame duration for the GPU rendering is added to the prediction. The last step of the prediction adds the duration of the scan-out, which is again retrieved from SteamVR. Finally, the predicted time is added to the initially measured time, which in sum makes up the stimulus onset time. The complete framework for the prediction is illustrated in Fig S3.

Note that the last function returns the time from VSync until the midpoint of the stimulus presentation instead of the time of the actual onset, i.e., the very first moment when the displays light up. This results from the default behavior of SteamVR’s pose prediction. Therefore, the predicted time is effectively another frame duration resulting in an over-prediction of about 1 ms.

Furthermore, the refresh rate returned by SteamVR, which forms the basis of the calculation for the frame duration, is 90 Hz for the HTC Vive. However, the actual refresh rate that we have measured in Experiment 1 (89.53 Hz) was slightly lower, which results in an under-prediction of each of the three steps of about 58 µs. Taken together, the proposed method does not predict the stimulus onset exactly but results in an over-prediction of about 826 µs, when used with the HTC Vive.

In principle, the over-prediction could be corrected by subtracting 826 µs from the result. However, we decided not to correct this marginal over-prediction due to the following reason. The algorithm for the prediction is completely based on functions provided by OpenVR without any hardcoding. This implementation, at least in principle, should enable experimenters to use the method also with HMDs other than the HTC Vive, as long as they use SteamVR as the runtime.
